# Primary synovial osteochondromatosis of a subdeltoid bursa

**DOI:** 10.4103/0019-5413.58613

**Published:** 2010

**Authors:** Anil Kumar, Arvind Aggarwal, Virender K Sahni

**Affiliations:** Department of Orthopedics, Maharaja Agrasen Hospital, Punjabi Bagh, New Delhi, India

**Keywords:** Subdeltoid bursa, synovial osteochondromatosis, extraarticular synovial osteochondromatosis

## Abstract

Primary synovial osteochondromatosis (SOC) is known to be intra-articular and wherever it is observed outside a synovial joint, it is associated with the involvement of the nearby joint. Primary SOC has not been reported to involve a subdeltoid bursa. We present a case of a 52-year-old woman having a large number of loose bodies in a large tumor in the subdeltoid bursa. The swelling was first noticed by the patient 2 years back. Plain roentgenogram revealed soft tissue swelling only with no areas of calcification. On MRI, multiple nonosseous loose bodies were visualized in the bursa deep to the deltoid muscle. A surgical excision of subdeltoid bursa was done. A biopsy confirmed it to be cartilaginous loose bodies in synovial lining sugestive of metaplastic transformation of the synovial tissue.

## INTRODUCTION

Synovial osteochondromatosis (SOC) is a benign metaplastic proliferative disorder of the synovium^1^–^5^ which affects subintimal fibroblasts in synovial joints,^4^,^6^,^7^ tendons, and bursae (involving articular or tendon sheath synovial membranes) in which multiple nodules of cartilage are produced.^3^,^5^,^8^ Many of the nodules subsequently become detached from the synovial membrane and float in the joint.^9^ The synovial fluid nourishes the loose bodies which can remain viable and increase in size.^2^,^3^,^7^,^10^ It is a rare disease and rarely affects the shoulder joint; it has male predominance affecting knee,^11^–^13^ elbow,^8^,^14^ hip,^10^,^15^–^19^ and ankle joints (in order of frequency) in the fourth/fifth decade of life.^4^,^7^ It presents with a stiffness of the joint and dull ache. The intra-articular loose bodies obstruct the joint mobility and the joint may get locked in a particular position. Theoretically, SOC may affect any synovial joint^3^,^6^ and may lead to a very big tumor sufficient to obstruct range of motion.

In respect to shoulder joint primary SOC has been reported in the subacromial bursa, subclavicular region and along the brachial plexus, but all are associated with the involvement of the glenohumeral joint.^20^–^23^ We present this case because it was completely extra-articular (below the bulk of the deltoid muscle) without involving the shoulder joint.

## CASE REPORT

**Figure 1:**
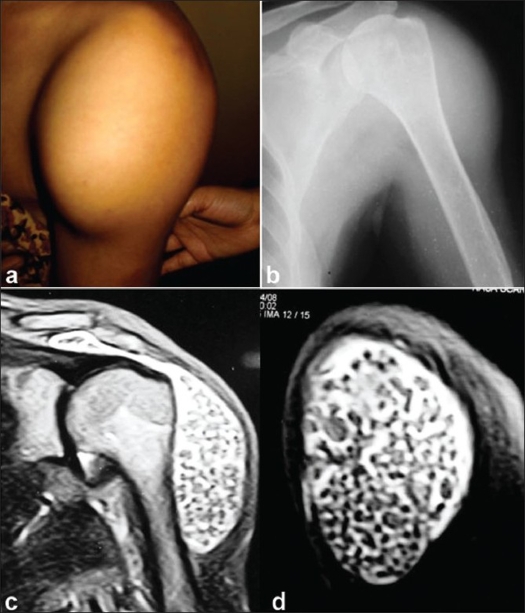
(a) A clinical photograph showing big swelling on the outer aspect of the left shoulder and left upper arm (b) X-ray left shoulder showing a soft tissue swelling in the deltoid region; no point/area of calcification visible in the swelling (c) MRI coronal section through the shoulder joint shows multiple nonossified loose bodies deep into the deltoid muscle with subacromial extension. No loose body below the rotator cuff (d) Another MRI sagittal section through the material of the swelling, showing a large number of loose bodies deep into the deltoid muscle; none is ossified

A 52-year-old woman presented with a swelling over the outer aspect of her left shoulder and proximal part of her left arm which she had first noticed 2 years ago. The swelling was obstructing the movements of her affected shoulder and was cosmetically unacceptable [Figure 1a]. She had no history of trauma or fever. Her appetite was not affected, and there had been no weight loss. The globular swelling was painless and measured approximately 8 inches in diameter. No tenderness or warmth, but some pseudofluctuation was observed, along with restriction of all movements except adduction. No abnormality was detected in central nervous, cardiovascular, respiratory, genitourinary, or gastrointestinal systems. She was postmenopausal with no gynecological problem. Routine preoperative investigations (including liver function and renal function tests) were within the normal limit. Serological investigation for rheumatoid arthritis was negative. Plain roentgenogram revealed a soft tissue swelling only [Figure 1b]. There were no areas of calcification.

On MRI, multiple nonosseous loose bodies were visualized in the bursa deep to the deltoid muscle. These spread up to the subacromial space as well as the anterior and posterior aspects of the shoulder. These appeared to remain outside the rotator cuff [Figure 1c and d]. Aspiration returned the nonhemorrhagic synovial fluid with no sign of tuberculosis or other chronic infective pathology.

**Figure 2:**
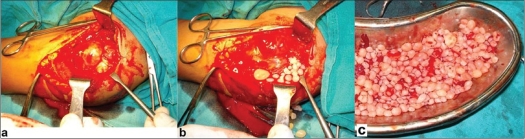
Per-operative photograph showing (a) synovial sheath is visible from under the deltoid muscle which is retracted laterally (incision in line of the deltopectoral groove); (b) cartilaginous loose bodies coming out of the incidentally ruptured synovial bag; (c) innumerable loose bodies after removal

**Figure 3:**
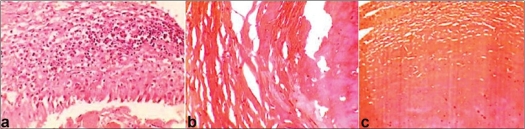
Histopathological micro-photograph: (a) black arrow denotes increased vascularity in the area of inflammation (granulation tissue); green arrow shows hyperplastic synovial lining; and blue arrow shows the early stage of development of the cartilaginous loose body which is still inside (b) chondrocytes in the hyaline cartilage (the blue thick arrow shows hyalinized tissue) (c) chondrocytes in lacunae inside the newly formed cartilage

The tumor was exposed through a linear incision over the deltopectoral groove. A large protruding synovial membrane was visible under the deltoid muscle [Figure 2a]. The synovial sheath was adherent to the surrounding structures. Innumerable pea-size cartilaginous loose bodies were extruded on an incidental rupture of the sheath [Figure 2b]. All the loose bodies were taken out and the whole bursal sheath was removed from below the deltoid and from the subacromial space [Figure 2c]. There was no connection of synovial bursa to the joint and all loose bodies remained external to the rotator cuff. A histopathological study revealed the early stages of the development of cartilaginous loose bodies in the synovial lining, suggestive of metaplastic transformation of the synovial tissue [Figure 3a and b]. Areas below the synovial lining contained a large number of mononuclear cells and a fair number of small blood vessels. This was consistent with chronic inflammation. In the cut-section of the loose body, chondrocytes were visible inside the newly formed cartilage [Figure 3c].

By 2 weeks, the wound was well healed and the patient recovered a full range of motion of the affected shoulder. At 1 year follow-up she does not have any recurrence.

## DISCUSSION

Osteochondromatosis can be primary or secondary. In secondary osteochondromatosis, the presence of free osteochondral bodies is due to osteoarthritis, trauma-causing osteochondral fractures, or osteochondritis dissecans. Primary SOC is not associated with frank osteoarthritis or synovitis^2^ and the source of the osteochondral loose bodies is the benign synovial neoplasia.^22^ Both the synovial membrane and articular cartilage develop from the same mesenchymal tissue, and this tissue arises from embryonic rests.^9^ Jones,^11^ in 1927, showed microscopically that the formation of these cartilaginous and osteocartilaginous bodies follow the same stages that occur in the embryonic formation of cartilage. The process is peculiar in that the bodies are formed from the synovial membrane instead of the articular surface, as in the case of osteochondritis dissecans and at times in the case of osteoarthritis. The bodies formed in SOC are composed of organized tissues, and are distinct from the unorganized tissues of “corpora oryzoidea,” or “rice bodies.”^2^

Mukerjiea^20^ reported SOC of the shoulder joint in a 12-year-old boy. Buess and Friedrich^21^ reported a case of a 22-year-old man with SOC of the shoulder joint. Recently, Antonogiannakis *et al*.^22^ reported a case of osteochondromatosis of the subacromial bursa in a 72-year-old retired military officer.

It is a rare disease^7^ and it rarely affects the shoulder joint.^3^,^5^,^7^,^9^,^15^–^28^ Multiple loose bodies are formed which get detached from the synovial membrane and may float in the synovial fluid inside the joint. It has been demonstrated in multiple studies in the shoulder^12^–^14^ but is never limited to the subdeltoid bursa. In our case, a large number of nonossified cartilaginous loose bodies were found in the synovial membrane of the subdeltoid bursa. These surrounded the shoulder but remained extra-articular.

Only when the bodies are calcified or ossified are they visible on plain roentgenograms,^17^ but the number is always greater than one would suspect from the film. Air or double contrast arthrography may be necessary to visualize the nonossified chondromatous bodies.^16^ But MRI could visualize almost all such bodies. Mussey and Henderson concluded that negative findings on microscopic examination of the synovial membrane do not necessarily negate a diagnosis of osteochondromatosis, for the process might have completed its cycle and the membrane might have resumed its normal appearance.^3^

Loose bodies must be removed early to halt further damage to articular surfaces.^10^,^15^,^19^ Synovial osteochondromatosis does not resolve spontaneously^7^ and complications like degenerative osteoarthritis,^10^ joint subluxation,^10^ and bursitis^8^ are not uncommon. The patient should be forewarned that a certain amount of degenerative arthritis is already present and may cause residual symptoms.^11^ It is controversial whether to perform complete synovectomy along with excision of all communicating bursae,^13^,^15^ because recurrences are common^11^,^15^,^19^ irrespective of the extent of the excision.^27^ Therefore, many authors favor removal of all loose bodies with subtotal synovectomy.^4^,^17^,^20^

Though suspected in some observations,^13^ sarcomatous degeneration of the synovial chondromatosis has not been proved.

## References

[1] Henderson MS, Joxis HT (1923). Loose bodies in joints and bursae due to synovial osteochondromatosis. J Bone Joint Surg.

[2] Jones HT (1924). Loose body formation in synovial osteochondromatosis with special reference to the etiology and pathology. J Bone Joint Surg.

[3] Mussey RD, Henderson MS (1949). Osteochondromatosis. J Bone Joint Surg Am.

[4] Jeffreys TE (1967). Synovial chondromatosis. J Bone Joint Surg Br.

[5] Dorfman HD (1998). Czerniak: Synovial chondromatosis. In synovial lesions. In “Bone Tumors”.

[6] Milgram JW (1977). Synovial osteochondromatosis: A histopathological study of thirty cases. J Bone Joint Surg Am.

[7] Murphy FP, Dahlin DC, Sullivan CR (1962). Articular Synovial Chondromatosis. J Bone Joint Surg Am.

[8] Matsumoto K, Hukuda S, Fujita M, Kakimoto A, Tachibana S (1996). Cubital bursitis caused by localized synovial chondromatosis of the elbow: A case report. J Bone Joint Surg Am.

[9] Turek SL (1984). Diseases of joints. In “Orthopaedics: Principles and their application”.

[10] Hardacker J, Mindell ER (1991). Synovial chondromatosis with secondary subluxation of the hip. A case report. J Bone Joint Surg Am.

[11] Jones HT (1927). The Histogenesis of cartilage as shown in chondromatosis of the knee joint. J Bone Joint Surg.

[12] Dunn AW, Whisler JH (1973). Synovial chondromatosis of the knee with associated extracapsular chondromas. J Bone Joint Surg Am.

[13] Milgram JW, Addison RG (1976). Synovial osteochondromatosis of the knee. chondromatous recurrence with possible chondrosarcomatous degeneration. J Bone Joint Surg Am.

[14] Kamineni S, O'Driscoll SW, Morrey BF (2002). Synovial osteochondromatosis of the elbow. J Bone Joint Surg Br.

[15] Lim SJ, Chung HW, Choi YL, Moon YW, Seo JG, Park YS (2006). Operative treatment of primary synovial osteochondromatosis of the hip. J Bone Joint Surg Am.

[16] Bloom R, Pattinson JN (1951). Osteochondromatosis of the hip joint. J Bone Joint Surg Br.

[17] McIvor RR, King D (1962). Osteochondromatosis of the hip joint. J Bone Joint Surg Am.

[18] Okada Y, Awaya G, Ikeda T, Tada H, Kamisato S, Futami T (1989). Arthroscopic surgery for synovial chondromatosis of the hip. J Bone Joint Surg Br.

[19] Lim SJ, Park YS (2007). Operative treatment of primary synovial osteochondromatosis of the hip. J Bone Joint Surg Am.

[20] Mukerjiea SK (1968). Synovial chondromatosis in shoulder joint. Proc Roy Soc Med.

[21] Buess E, Friedrich B (2001). Synovial chondromatosis of the glenohumeral joint: A rare condition. Arch Orthop Trauma Surg.

[22] Antonogiannakis E, Yiannakopoulos CK, Mataragas I (2008). Osteochondromatosis of the subacromial bursa arthroscopic treatment of a case and review of the literature. EEXOT.

[23] Sim FH, Dahlin DC, Ivins JC (1977). Extra-articular synovial chondromatosis. J Bone Joint Surg Am.

[24] Wakhlu A (2004). Synovial osteochondromatosis. J Indian Rheumatol Assoc.

[25] Wilmoth CL (1941). Osteochondromatosis. J Bone Joint Surg Am.

[26] Milgram JW, Pease CN (1980). Synovial osteochondromatosis in a young child: A case report. J Bone Joint Surg Am.

[27] Maurice H, Crone M, Watt I (1988). Synovial chondromatosis. J Bone Joint Surg Br.

[28] Shanbhag A, Balakrishnan C, Bhaduri A (2004). Primary synovial osteochondromatosis. J Indian Rheumatol Assoc.

